# The diagnostic and prognostic value of *CHFR* hypermethylation in colorectal cancer, a meta-analysis and literature review

**DOI:** 10.18632/oncotarget.19408

**Published:** 2017-07-20

**Authors:** Zhulei Sun, Juncai Liu, Hong Jing, Shu-Xiao Dong, Jiang Wu

**Affiliations:** ^1^ Department of Pathology, Huaihe Hospital, Henan University, 8 Bao Bei Lu, GuLou Qu, Kaifeng 475000, China; ^2^ Department of Radiotherapy, Huaihe Hospital, Henan University, Kaifeng 475000, China; ^3^ Department of Gastrointestinal Surgery, Linyi People’s Hospital, Linyi 276001, Shandong, China

**Keywords:** CHFR, methylation, biomarker, CRC, colorectal carcinoma

## Abstract

The Checkpoint with Forkhead-associated and Ring finger domains (*CHFR*) is a mitotic checkpoint and tumor-suppressor gene, its loss contributes tumorigenesis of epithelial cancers including colorectal carcinoma (CRC). The diagnostic and prognostic value of *CHFR* promoter hypermethylation in CRC remains unclear. This study aimed to conduct a meta-analysis and literature review and investigate clinicopathological significance of *CHFR* promoter hypermethylation in CRC. The following online database were used: PubMed, EMBASE, and Web of Science up to March 2017. Odds Ratios (OR) and Hazard Ratios (HR) with 95% corresponding confidence intervals (CIs) were calculated. A total of seven relevant articles were available for meta-analysis which included 966 patients. The frequency of *CHFR* promoter hypermethylation significantly increased in CRC compared to normal colorectal mucosa tissue, pooled OR was 8.35, *p* < 0.00001. *CHFR* promoter hypermethylation was not significantly correlated to stage, OR was 1.16, *p* = 0.63. However, *CHFR* promoter hypermethylation was more frequently observed in CRC with positive lymph nodes metastasis than CRC with negative lymph nodes metastasis, OR was 0.46, *p* = 0.03. Additionally *CHFR* promoter hypermethylation was significantly related to poor overall survival in patients with CRC, HR was 0.62, *p* = 0.008. Based on these results, tumor *CHFR* promoter hypermethylation is not only a diagnostic biomarker for CRC, but also a prognostic marker. *CHFR* promoter hypermethylation is significantly associated with worse overall survival in patients with CRC. Our data suggested that CHFR could be a potential drug target for development of demethylation treatment for patients with CRC.

## INTRODUCTION

Colorectal cancer (CRC) is the third most frequently diagnosed malignancy among males and females, and the third leading cause of cancer-related mortality in the United States [1, 2]. CRC is largely asymptomatic until alarm features develop to advanced stages [3]. Traditional CRC staging takes into account the depth of invasive growth, the histological differentiation grade, and the presence of metastasis in lymph nodes as well as in distant organs. This staging system assesses the anatomic tumor extent only, does not consider the various patients, tumor and environmental factors that influence prognosis. Thus, it is crucial to identify molecular biomarkers for prediction of prognosis and development of new drug target. Checkpoint with Forkhead-associated and Ring finger domains (*CHFR*) is a G2/M checkpoint gene that has lately been reported by Scolnik et al. [4]. This protein functions as an ubiquitin ligase that consists of a forkhead and a RING finger domain [5]. CHFR mediates delay in M-phase entry, when cells are challenged with an inhibitor of microtubule assembly. CHFR is a ligase that ubiquinates and inhibits Polo-like kinase 1 (Plk 1 kinase), leading to delay in activation of cdc2 kinase, the key regulator of G2/M phase entry [6]. *CHFR* promoter hypermethylation was observed in various human cancers including 20% in Non-Small Cell Lung Cancer (NSCLC) [7], 30% in esophageal cancers [8], and 40% in CRC [9]. However, the role of *CHFR* methylation in the progression and prognosis of CRC remains unclear due to the small size sample of individual studies. The aim of present study is to investigate the diagnostic and prognostic value of *CHFR* methylation in the tumorigenesis and progression of CRC with a meta-analysis which increases the sample size and thus the power.

## RESULTS

### Identification of relevant studies

**Figure 1:**
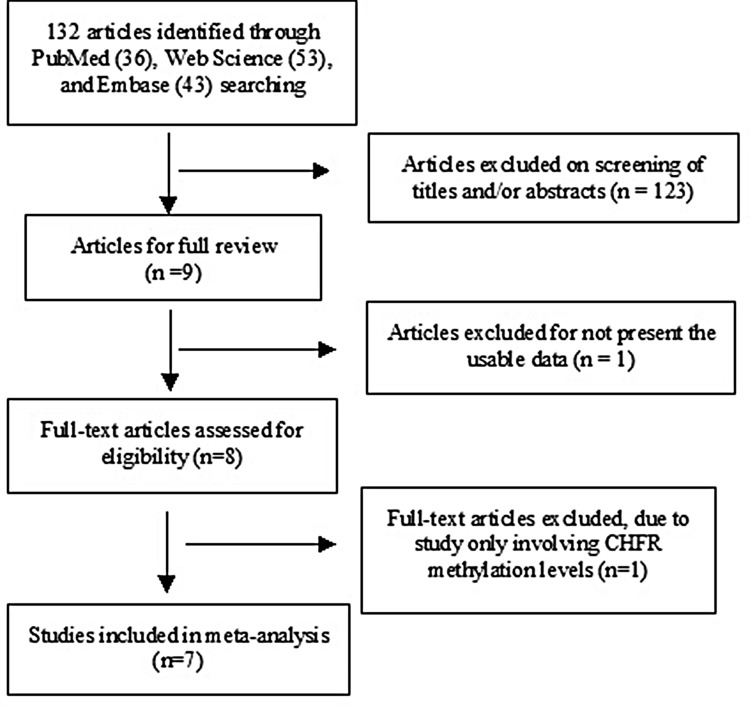
Schematic flow diagram for selection of included studies

A total of 132 references were identified in PubMed, EMBASE, and Web of Science databases. Most of them were excluded after reviewing the abstracts. Seven articles were included in the review (Figure 1).

### Study characteristics

Seven articles were published from 2005 to 2014. A total of 966 CRC patients from the United States, the United Kingdom, Finland, Japan and the Netherlands were included. Their basic characteristics are presented in Table 1.

**Table 1:** Basic characteristics of the included studies

Study	Country	Patients	Methods	Primary Aim	Methylation site	CHFR expression
Cleven et al. 2014 [24]	Netherland	468	MSP	Determine the prognostic role of CHFR in stage II microsatellite stable colorectal cancer	Promoter, CpG	-
Tanaka et al. 2011 [25]	USA	82	Pyrosequencing/Immunochemistry	Study the association of CHFR promoter methylation with disease recurrence in advanced colon cancer	Promoter, CpG	+
Leong et al. 2011 [26]	UK	70	MSP	Investigate methylation profile of rectal cancer and identify novel markers	Promoter, CpG	-
Joensuu et al. 2008 [27]	Finland	148	MS-MLPA	Study epigenetic signatures of familial cancer in different tumor types and family categories.	Promoter, CpG	-
Derks et al. 2006 [28]	Netherlands	36	MSP	Study the timing of promoter methylation and relationship with mutations and chromosomal alterations in colorectal carcinogenesis.	Promoter, CpG	-
Morioka et al. 2006 [29]	Japan	98	MSP/RT-PCR	Study aberrant methylation of CHFR in colorectal cancer	Promoter	-
Brandes et al. 2005 [30]	Netherlands	64	MSP	Study the correlation of CHFR hypermethylation with microsatellite instability phenotype.	Promoter	-

MSP: methylation-specific PCR, MS-MLPA: Methylation-specific multiplex ligation-dependent probe ampliification.

### Study quality, sensitivity analyses and publication bias

The quality of individual study was evaluated by using the Newcastle Ottawa Quality Assessment Scale (NOQAS). The score of six studies were greater than or equal to seven that indicated relatively high quality (data not shown), one of them scored six points. A sensitivity analysis was performed by omitting one study from the meta-analysis at a time, the overall results stayed stable (Supplementary Figures 1 and 2). The largely symmetric funnel charts indicated no publication biases in the meta-analysis (Figure 6A–6D).

**Figure 2:**
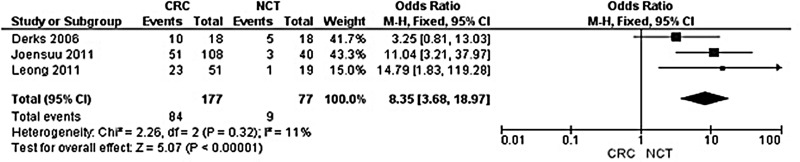
Forest plot for *CHFR* promoter hypermethylation in CRC and normal colorectal tissue

**Figure 3:**
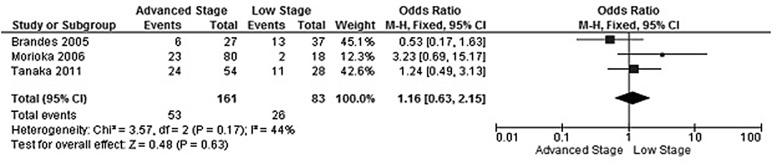
Forest plot for *CHFR* promoter hypermethylation in stage III/IV and stage I/II of CRC

**Figure 4:**
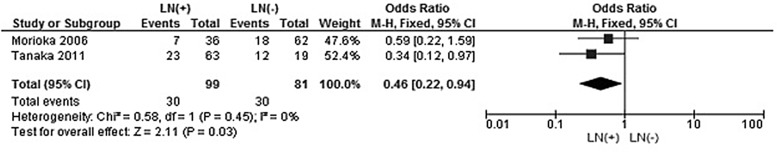
Forest plot for *CHFR* promoter hypermethylation in CRC patients with different lymph node metastasis status

**Figure 5:**
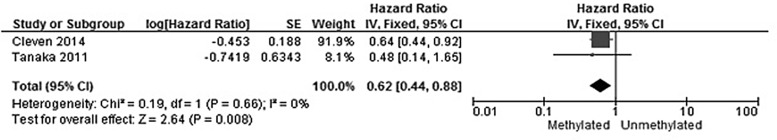
Forest plot for the correlation between *CHFR* promoter hypermethylation and the overall survival of patients with CRC

**Figure 6:**
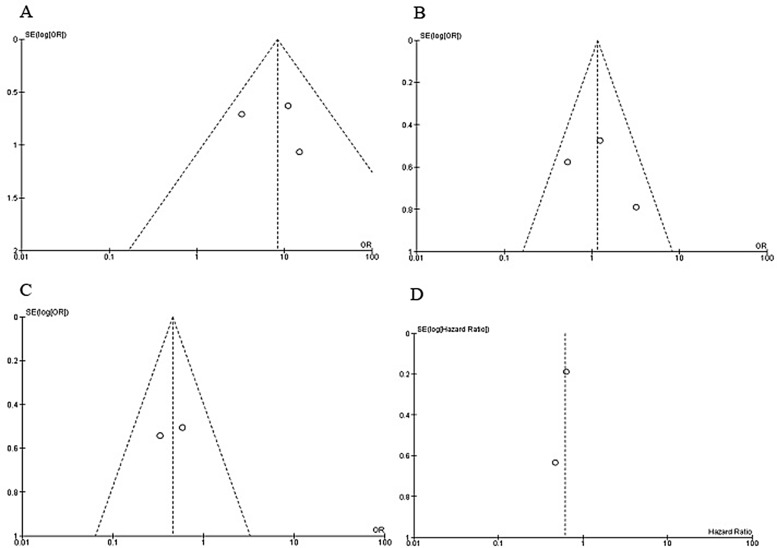
Funnel plot for publication bias (**A**) *CHFR* promoter hypermethylation in CRC and normal colorectal tissue. (**B**) *CHFR* promoter hypermethylation in stage III/IV and stage I/II of CRC. (**C**) *CHFR* promoter hypermethylation in CRC patients with different lymph node metastasis status. (**D**) The correlation between *CHFR* promoter hypermethylation and the overall survival of patients with CRC. S.E., standard error; Area of the circle represents the weight of individual study.

### The association of *CHFR* promoter hypermethylation with clinicopathological characteritics

The rate of *CHFR* promoter hypermethylation in CRC was significantly higher than in normal colorectal mucosa tissue, the OR was 8.35 with 95% CI 3.68–18.97, z = 5.07, *p* < 0.00001 (Figure 2). *CHFR* promoter hypermethylation was not significantly associated with stages, OR was 1.16 with 95% CI 0.63–2.15, z = 0.48, *p* = 0.63 (Figure 3). *CHFR* promoter hypermethylation was significantly associated with lymph nodes metastasis status, OR was 0.46 with 95% CI 0.22–0.94, z = 0.36, *p* = 0.03 (Figure 4). *CHFR* promoter hypermethylation was significantly related to the poor overall survival in patients with CRC, HR was 0.62 with 95% CI 0.44–0.88, z = 2.64, *p* = 0.008 (Figure 5)

## DISCUSSION

Precise staging of CRC is crucial for optimal disease treatment. The traditional tumor-node-metastasis (TNM) classification system is the most reliable indicator of prognosis and provides the guideline for treatment plan of CRC. Adjuvant therapy is desirable for some of stage II and all stage III CRC patients. With the TNM classification system, the outcome can be different in patients with the same stage of CRC, thus identification of a subgroup with molecular data will probably allow better patient selection for adjuvant chemotherapy. Prior studies indicated that CRC is characterized by epigenetic or genetic abnormalities of gene which controls the progression and prognosis of cancer [11–14]. Inactivation of tumor suppressor genes by promoter hypermethylation has been implicated in CRC [15]. Previous studies showed different rate of *CHFR* promoter hypermethylation in CRC patients. In present study we pooled three studies and analyzed the rate of *CHFR* promoter hypermethylation in CRC patients, found that *CHFR* promoter was 8.36 times more frequently hypermethylated in CRC patients compared to normal colorectal tissue, suggesting that *CHFR* promoter hypermethylation is a promising biomarker for diagnosis of CRC. Moreover, *CHFR* could be a novel target for development of personalized cancer treatment.

*CHFR* is a mitotic checkpoint and tumor suppressor gene, which is silenced in a variety of human cancers, mostly by promoter CpG island methylation [17]. Scolnick and Halazonetis first described the inactivation of CHFR in neuroblastoma and colorectal cancer cell lines [4]. In contrast to wild-type cancer cells, *CHFR* methylation led to an increased mitotic index in these cell lines treated with inhibitor of microtubule assembly. Whereas the mitotic index was decreased by re-expression of CHFR in the cell lines after demethylation with 5-aza-2′-deoxycytidine l [18]. In addition, the potential role of hypomethylation agents, such as azacitidine (AZA), has been demonstrated in reversing the effects of hypermethylation in solid tumors [16]. Therefore, *CHFR* demethylation is a promising personalized therapy, further validation is required in CRC patients with *CHFR* hypermethylation.

Previous data indicated that CHFR localizes to the mitotic spindle through an interaction with beta-tubulin and Translationally Controlled Tumor-associated Protein (TCTP), a protein related to microtubule stabilization [19]. Interruption of the spindle results in CHFR to release from TCTP and the mitotic spindle, which will activate the signaling pathways and finally delay cell cycle progression [20]. Oh et al. reported that, *in vitro*, CHFR binds and represses histone deacetylase 1 (HDAC1) leading to upregulating cycline-dependent kinase inhibitor 1 and the metastasis suppressors, KAI1 as well as Cadherin-1 [21].

Previous evidence indicated *CHFR* hypermethylation was associated with the risk of lymph node involvement and overall survival, but the change was not significant. We pooled the individual studies and performed a meta-analysis. Our pooled data showed *CHFR* promoter hypermethylation significantly increased the risk of lymph node metastasis in CRC patients. In addition, *CHFR* promoter hypermethylation was significantly correlated to poor prognosis in patients with CRC, indicating *CHFR* hypermethylation could be a prognostic predictor. Oh et al. evaluated the expression level of CHFR and HDAC1 in human prostate (PC-3) or breast (MCF7) cancer cells. The group reported that the expression of all potential substrates including HDAC1, Plk1 and Aurora A were higher in metastatic cancer cells than in normal cells. HDAC1 may repress expression of the metastasis suppressor gene KAL1 and the invasion suppressor gene E-cadherin [22, 23]. Thus the prognosis was poor in CRC patients with *CHFR* promoter hypermethylation.

Sensitivity analysis indicated that there was not an individual study was found to be significantly biasing the pooled result. There was no evidence of publication bias found. The meta-analysis has potential limitations: 1). Present findings were based on individual unadjusted ORs, while further evaluation needs to be finished by other potential risk factors. 2). All the included studies are observational studies that selection bias and publication bias may exist, since positive results may be more likely to be published than negative results. 3). The searching strategy was limited to articles published in English and Chinese, while studies published in other language were excluded in present study. 4). A small number of patients were evolved in some of included studies so that the confidence intervals within studies was large, this makes it difficult to estimate the real effect. 5). Finally, further studies with large numbers of subjects are essential to validate the results we obtained.

In summary, *CHFR* hypermethylation is not only a diagnostic biomarker for CRC, but also a prognostic marker. The higher level of *CHFR* methylation is significantly associated with worse overall survival compared to patients with a lower level of *CHFR* tumor DNA methylation. Our data suggested that *CHFR* could be a promising therapeutic target of development of demethylation treatment for patients with CRC.

## MATERIALS AND METHODS

### Search strategy and selection criteria

A systematic and comprehensive literature searches was performed for related studies published before March 2017 in the PubMed, EMBASE, Web of Science with no limit set for date and language of publication using the search terms: “colorectal carcinoma”, “CRC”, “methylation”, and “CHFR, or Checkpoint with Forkhead-associated and Ring finger domains”. There were 36 studies were identified from PubMed, 43 studies from EMBASE, 53 studies from Web Science. A total of 132 studies were reviewed by article titles and abstracts.

After reviewing by titles and abstracts, individual studies were screened using the inclusion and exclusion criteria. The inclusion criteria are as following: 1) studies that evaluated *CHFR* hypermethylation in the primary CRC tissues, 2) studies revealed the relationship between *CHFR* hypermethylation and CRC clinicopathological features, 3) *CHFR* hypermethylation examined by polymerase chain reaction (PCR). The exclusion criteria included the following: 1) case reports, conference abstracts, reviews, letters, editorials, expert opinion, 2) all studies using cell lines, human xenografts, and studies *in vitro*/*ex vivo* were also excluded. The search process was conducted independently by two reviewers (ZS and JL), discrepancies were discussed and resolved by the third reviewer (HJ). Forward and backward citation chasing of each included article was conducted. The most complete study was chosen to avoid duplication if same patient populations were reported in several publications. Seven articles were eligible for inclusion in this meta-analysis.

### Data extraction and methodological assessment

Two authors (JL and SD) independently reviewed and extracted following data: last name of the first author, year of publication, country where the study was conducted, number of CRC cases, clinicopathological parameters, cancer TNM (tumor node metastasis) stage, methylation detection method, methylation rate. The detailed information of seven relevant articles was listed in Table 1. Heterogeneity of investigation was evaluated to determine whether or not the data of various studies could be analyzed for a meta-analysis.

For the methodological evaluation of the studies, three investigators (HJ, SD, and JW) read through each publication independently, and they assessed and scored them according to the REMARK guidelines and ELCWP quality scale. Three reviewers provided the quality scores and compared them, and then they reached a consensus value for each item.

The quality of each study was independently scored by three reviewers according the Newcastle Ottawa Quality Assessment Scale (NOQAS). These scales were utilized to allocate a maximum of nine points for the quality of selection, comparability, exposure, and outcomes for study participants, and a score ≥ 7 was considered as a good quality.

### Statistical analysis

Analysis was conducted using Review Manager 5.2 (Cochrane Collaboration, Oxford, UK). The pooled odds ratios (ORs) with its 95% confidence intervals were calculated. Heterogeneity among studies was estimated using the Cochran’s Q statistic and *I*^2^ tests [10]. The *I*^2^ statistics was used to examine the difference for between study variability due to heterogeneity rather than chance, with a range from 0 to 100 percent. When heterogeneity (*I*^2^) was less than < 50%, a fixed effect model was used to calculate parameters. If there was substantial heterogeneity (*I*^*2*^ values ≥ 50%), a random-effect model was used to pool data and attempt to identify potential sources of heterogeneity based on subgroup analyses. The analysis was performed to compare the frequency of *CHFR* methylation between CRC and normal colorectal tissue. The frequency of *CHFR* hypermethylation was compared in different tumor characteristics. The pooled ORs were estimated for the correlation between *CHFR* hypermethylation and clinicopathological features. *P* values tailed less than 0.05 were considered statistically significant. Publication bias is what occurs whenever the research in the published literature is systematically unrepresentative of population of completed studies. Funnel plots were used for detection of publication bias.

## SUPPLEMENTARY MATERIALS FIGURES


