# Supplementary material for: The diagnostic and prognostic value of CHFR hypermethylation in colorectal cancer, a meta-analysis and literature review
Source: Oncotarget. 2017 Jul 20;8(51):89142–8. doi: 10.18632/oncotarget.19408 (PMC5687676; doi:10.18632/oncotarget.19408)
Supplement: Supplementary file 1 [file oncotarget-08-89142-s001.pdf]

The diagnostic and prognostic value of *CHFR* hypermethylation in colorectal cancer, a meta-analysis and literature review

SUPPLEMENTARY MATERIALS

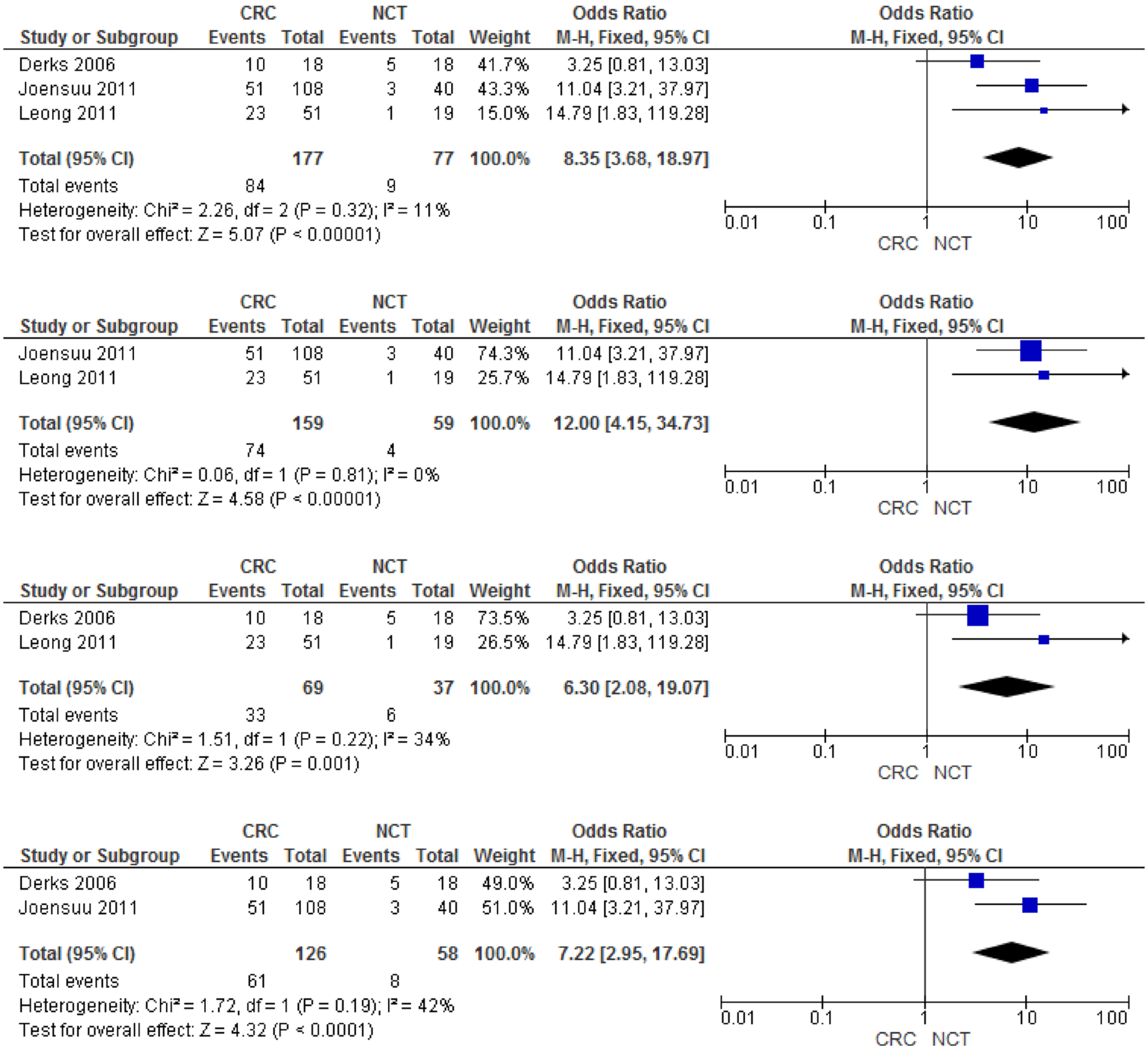

Supplementary Figure 1: The sensitivity analysis of *CHFR* promoter hypermethylation in CRC and normal colorectal tissue.

Study or Subgroup	Advanced Stage Events	Advanced Stage Total	Low Stage Events	Low Stage Total	Weight	Odds Ratio M-H, Fixed, 95% CI
Brandes 2005	6	27	13	37	45.1%	0.53 [0.17, 1.63]
Morioka 2006	23	80	2	18	12.3%	3.23 [0.69, 15.17]
Tanaka 2011	24	54	11	28	42.6%	1.24 [0.49, 3.13]
**Total (95% CI)**		**161**		**83**	**100.0%**	**1.16 [0.63, 2.15]**
Total events	53		26			

Heterogeneity: Chi² = 3.57, df = 2 (P = 0.17); I² = 44%
Test for overall effect: Z = 0.48 (P = 0.63)

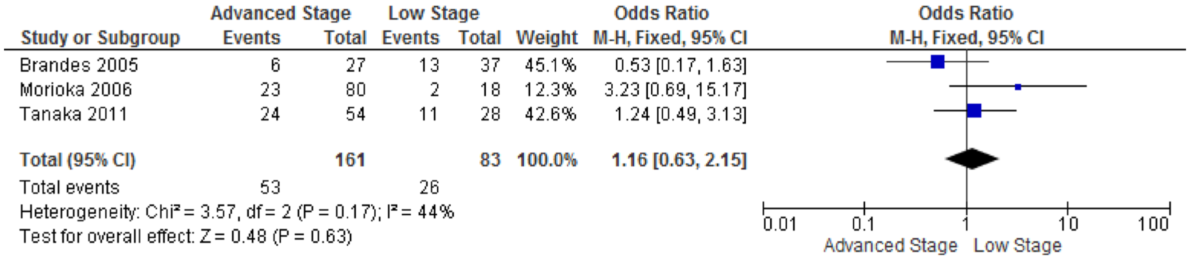

Study or Subgroup	Advanced Stage Events	Advanced Stage Total	Low Stage Events	Low Stage Total	Weight	Odds Ratio M-H, Fixed, 95% CI
Morioka 2006	23	80	2	18	22.4%	3.23 [0.69, 15.17]
Tanaka 2011	24	54	11	28	77.6%	1.24 [0.49, 3.13]
**Total (95% CI)**		**134**		**46**	**100.0%**	**1.68 [0.78, 3.65]**
Total events	47		13			

Heterogeneity: Chi² = 1.10, df = 1 (P = 0.29); I² = 9%
Test for overall effect: Z = 1.32 (P = 0.19)

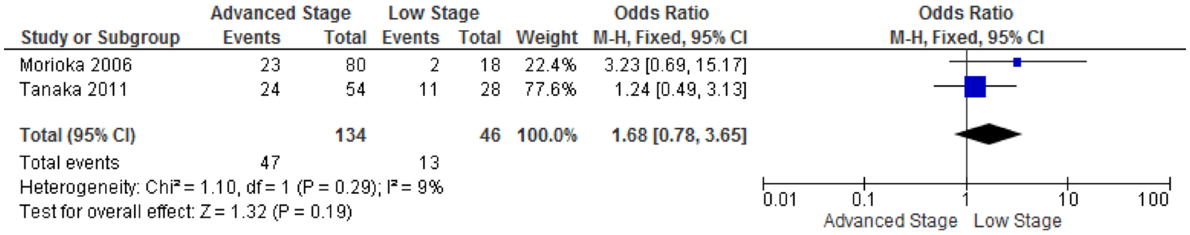

Study or Subgroup	Advanced Stage Events	Advanced Stage Total	Low Stage Events	Low Stage Total	Weight	Odds Ratio M-H, Fixed, 95% CI
Brandes 2005	6	27	13	37	51.5%	0.53 [0.17, 1.63]
Tanaka 2011	24	54	11	28	48.5%	1.24 [0.49, 3.13]
**Total (95% CI)**		**81**		**65**	**100.0%**	**0.87 [0.43, 1.76]**
Total events	30		24			

Heterogeneity: Chi² = 1.30, df = 1 (P = 0.25); I² = 23%
Test for overall effect: Z = 0.38 (P = 0.70)

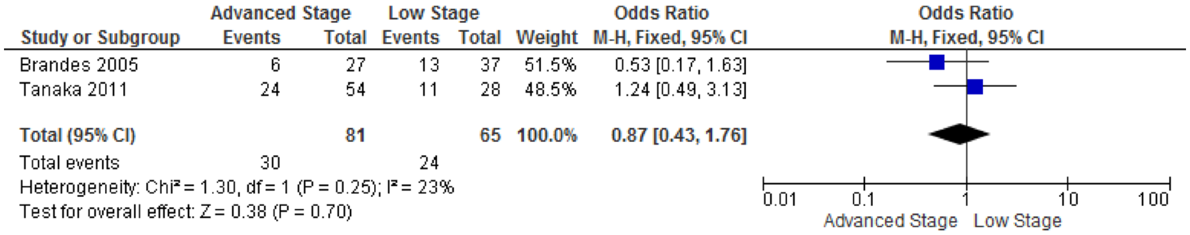

Study or Subgroup	Advanced Stage Events	Advanced Stage Total	Low Stage Events	Low Stage Total	Weight	Odds Ratio M-H, Fixed, 95% CI
Brandes 2005	6	27	13	37	78.6%	0.53 [0.17, 1.63]
Morioka 2006	23	80	2	18	21.4%	3.23 [0.69, 15.17]
**Total (95% CI)**		**107**		**55**	**100.0%**	**1.11 [0.49, 2.52]**
Total events	29		15			

Heterogeneity: Chi² = 3.49, df = 1 (P = 0.06); I² = 71%
Test for overall effect: Z = 0.24 (P = 0.81)

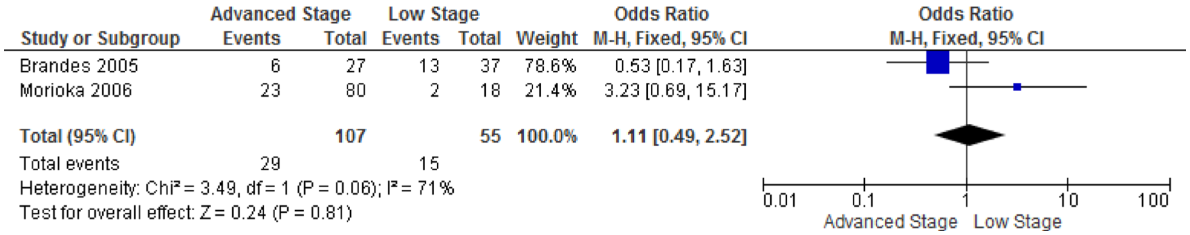

Supplementary Figure 2: The sensitivity of analysis of *CHFR* promoter hypermethylation in stage III/IV and stage I/II of CRC.
